# Effect of Various Surface Treatments on Wettability and Morphological Properties of Titanium Oxide Thin Films

**DOI:** 10.3390/ma15124113

**Published:** 2022-06-09

**Authors:** Ewelina Kuźmicz-Mirosław, Marcin Kuśmierz, Konrad Terpiłowski, Mateusz Śmietana, Mariusz Barczak, Magdalena Staniszewska

**Affiliations:** 1 SDS Optic S.A. Głęboka 39, 20-612 Lublin, Poland; emiroslaw@sdsoptic.pl; 2 Institute of Chemical Sciences, Faculty of Chemistry, Maria Curie-Sklodowska University, Maria Curie-Sklodowska Sq. 3, 20-031 Lublin, Poland; marcin.kusmierz@mail.umcs.pl (M.K.); konrad.terpilowski@mail.umcs.pl (K.T.); 3 Institute of Microelectronics and Optoelectronics, Warsaw University of Technology, Koszykowa 75, 00-662 Warszawa, Poland; msmietana@elka.pw.edu.pl

**Keywords:** titania, surface treatment, surface activation, hydroxylation, hydroxyl groups, wettability

## Abstract

The effect of three popular surface activation methods for a titanium oxide (titania) surface was thoroughly investigated to identify the most effective protocol for the enhancement of hydrophilicity. All the methods, namely H_2_O_2_ activation, UV irradiation and oxygen plasma treatment resulted in an enhanced hydrophilic titania surface, which was evidenced by the reduced contact angle values. To study in detail the chemical and morphological features responsible for the increased hydrophilicity, the treated surfaces were submitted to inspection with atomic force microscopy and X-ray photoelectron spectroscopy. The correlation between the treatment and titania surface hydroxylation as well as hydrophilic behavior have been discussed.

## 1. Introduction

Titania (titanium oxide, TiO_2_) has been widely used in many areas of life (pigment, food coloring, cosmetics, toothpastes) and technology (medicine, energy and biosensing) [1,2]. After the discovery of the phenomenon of photocatalytic splitting of water on a titania electrode under ultraviolet irradiation, a huge growth of research activities has been seen on various TiO_2_ materials, leading to many promising applications in areas of photovoltaics, photocatalysis and sensing [3,4]. Another interesting application of titanium dioxide is self-cleaning and self-sterilizing surfaces that can maintain their activity indefinitely as long as they are UV or even VL illuminated [5]. This is because when the titanium oxide film is illuminated with UV light, the contact angle is reduced to almost 0°. In fact, TiO_2_ is one of the few low-cost materials known to show this change in wettability under light illumination [6].

Applications of titania in biomedicine also gather significant attention owing to its excellent biocompatibility, unique photocatalytic properties, high chemical stability, and low toxicity [7]. Particularly, titania coatings are widely applied for improving the biocompatibility and bioactivity of implant materials or for the biosensing purposes, where a wide range of biomolecules can be immobilized to its surface and provide specific sensing functions [8]. In fact, the excellent biocompatibility of titanium itself is owed to a thin, several nm thick amorphous TiO_2_ film naturally formed on its surface, protecting titania from various external environments, which could deteriorate the properties of the resulting surface [9].

In reality, the adsorption of ubiquitous water on TiO_2_ surface always takes place [10], either as the molecular or dissociative form [11]. The latter plays an important role in further application of the material as it leads to the formation of surface hydroxyl groups. As a consequence of dissociative adsorption of water resulting in the cleavage of some O–H bonds, on a TiO_2_ surface, different kinds of hydroxyl groups are formed. The isolated and bridged hydroxyls are present on different sites depending on the specific titania crystal planes [12]. The fully hydroxylated TiO_2_ surface presents two types of hydroxyl groups: (i) a terminal OH group, bound to a surface Ti^4+^ site, which has five coordinates in respect to the lattice oxide ions, and (ii) a bridging OH group, bound to a Ti^4+^ site, which has four coordinates with respect to the lattice oxide ions [13]. Moreover, the adsorbed water can be further bonded to surface hydroxyls through strong hydrogen-bonding interactions, significantly affecting the final properties of titania, e.g., wettability, chemical composition and surface morphology [14]. It ultimately drives the covalent and non-covalent interactions with polymers, proteins, and impacts the immobilization of organic molecules [15,16,17]. Therefore, it is extremally important to functionalize the surface with a significant amount of hydroxyls for further tuning of the interactions between titania and immobilized materials. In many cases, the content of surface hydroxyl groups has to be significantly increased to assure efficient attachment of the targeted molecules to the hydroxylated surface. Thus, pre-activation of the titania surface is of high importance for many applications, particularly in biomedicine where such surface can be used, i.e., as scaffold for tissue regeneration [18]. In addition to the chemical changes, the surface experiences a change in topography having an impact on a wide range of physical and chemical properties, morphology and wettability. All these together determine the hydrophilic properties as more effective for cellular interaction.

In this work, we have selected, applied to titania and compared three popular methods of surface activation, namely oxygen plasma, UV light, and H_2_O_2_ activation. These methods were selected as we had previous experience (published and unpublished) of using plasma and H_2_O_2_ on silica glass slides, and we observed increased wettability after those treatments (e.g., [19,20]). Plasma treatment is a physicochemical process where atoms of high energy can remove or significantly modify a surface. When it is applied for a short time and with low energy delivered to atoms, it allows for avoiding damaging the titania surface. Oxygen plasma improves the wettability of the surfaces, efficiently oxidizes and removes contaminants [21]. Implementing the oxygen/air plasma results in creation Ti^3+^ and oxygen vacancies in TiO_2_ films [22]. As a result, the oxygen plasma treatment promotes the hydrophilicity of the TiO_2_ surface [23].

Additionally, UV light was chosen as it is known to increase the wettability of the TiO_2_ surface [24,25]. Exposition of the TiO_2_ surface to UV light results in the generation of highly reactive radicals that efficiently degrade nearby organic species [26]. UV activates only the light-available surface in contrast to plasma that can activate also UV light unavailable areas [27]. Of course, the efficacy of the plasma treatment is also pore size dependent, because the plasma cannot be generated in pores smaller than the mean free path [28]. The additional advantages of UV irradiation are photocatalytic sterilization and photo-induced superhydrophilicity (understood as complete wetting, i.e., water contact angles approaching 0 degrees) [29]. H_2_O_2_ oxidation is a classical wet route of surface activation. Hydrogen peroxide is a powerful oxidant, as demonstrated by the E° value of 1.76 V measured for the half-reaction H_2_O_2_ + 2 H^+^ + 2 e^−^ → 2 H_2_O [30]. Either TiO_2_ or H_2_O_2_ are not active when exposed to visible light, but the TiO_2_–H_2_O_2_ complex shows visible light sensitivity because of the peroxy-complex formation on the TiO_2_ surface [31]. H_2_O_2_ is sometimes used with other co-activators aiming to provide a better activation efficiency [32]. In the current investigation, plasma, UV, and H_2_O_2_-exposed TiO_2_ surfaces were compared to verify the impact of these methods on such surface properties as wettability, morphological and chemical features.

## 2. Materials and Methods

### 2.1. Materials

The following materials and reagents were used as received: titania-coated glass slides prepared according to the proprietary process of SDS Optic S.A., resulting in TiO_2_ thickness in the range of 50–100 nm, H_2_O_2_ (POCH, Gliwice, Poland), ethanol (POCH, Poland), and demineralized water of purity class 1.

### 2.2. Activation Treatments

Based on the parameters used previously in the literature, the following three activation methods were applied to the TiO_2_-coated glasses:

(i) Oxygen cold plasma activation—titania-coated glass slides were placed in a vacuum chamber of plasma cleaner (Pico, Diener Electronic, Ebhausen, Germany) and activated for one minute at 500 W power (using oxygen (pressure: 0.2 mbar, flow: 22 sccm)).
(ii) UV activation—titania-coated glass slides were inserted into the UV chamber (KW-4AC UV Curer, Chemat) and subjected to irradiation with the use of four UV lamps with a total power of 16 W for 2 h.
(iii) Titania-coated glass slide were immersed in 30 mL of 30% hydrogen peroxide. The Petri dish in which the activation was carried out was placed on a magnetic stirrer (RCT basic, IKA, Staufen im Breisgau, Germany) and gently stirred for 30 min.

### 2.3. Surface Characterization

X-ray photoelectron (XPS) spectra were collected using a multi-chamber UHV system (PREVAC, Rogów, Poland). Spectra were collected using a hemispherical R4000 electron analyzer, SAX-100 X-ray source (Al Kα, 1486.6 eV, 0.8 eV band) and XM 650 X-Ray Monochromator (0.2 eV band) with X-ray spot dimensions of 8 × 2 mm. The pass energy of the analyzer was set to 200 eV for survey spectra (with 500 meV step) and 50 eV for regions (with 100 meV step). The base pressure in the analysis chamber was 5 × 10^−9^ mbar. The X-ray monochromator was placed at a magic angle relative to analyzer axis; thus, no angular correction was required. Spectra processing was completed using CasaXPS v. 2.3.23PR-1.0 software, and Scofield cross-sections included in the software library were used. Due to the insulating nature of the samples, we used a flood gun for charge neutralization. All the spectra were corrected by setting the aliphatic carbon peak binding energy at 284.7 eV. For spectra deconvolution, a Shirley background and Gaussian–Lorenzian peak shapes were used (30% of Gaussian, aka GL(30) in CasaXPS). In O 1s, the spectra peak widths were restricted to 1.35 eV [33]. 

Scanning Electron Microscopy (SEM) images of the bare titania surface were collected with a Quanta 3DFEG (Thermo Fisher Scientific/FEI, Waltham, MA, USA) microscope with the accelerating voltage of 20 keV.

Surface topology experiments were carried out with Atomic Force Microscopy (AFM) on a NanoScope V instrument (Veeco, Plainview, NY, USA) equipped with a piezoscanner of a maximum scan range of 150 μm × 150 μm. A ScanAsyst-AIR-HR cantilever/tip (Bruker, Billerica, MA, USA) with a spring constant of 0.4 N/m, tip radius of 2–12 nm and resonance frequency of 100–160 kHz was used. The topography and amplitude/deflection images were obtained simultaneously. Taping mode was used for imaging the topography of the external surface of the TiO_2_ layers. NanoScope Analysis ver. 1.40 software was applied to AFM data processing and statistical analysis. Three images were collected per sample, each from a different part of the surface. Parameters S_q_, S_a_ and S_diff_ (discussed later in this paper) were averaged from all three images for each sample.

Water contact angles (CA) were measured in triplicate (for each of three replicates, the CA measurement was performed on both sides of the droplet) using the GBX contact angle meter controlled by Win Drop++ software. Then, 6 μL water droplets were gently settled on the surface using an automatic deposition system and immediately submitted to contact angle measurements.

## 3. Results

**Figure 1:**
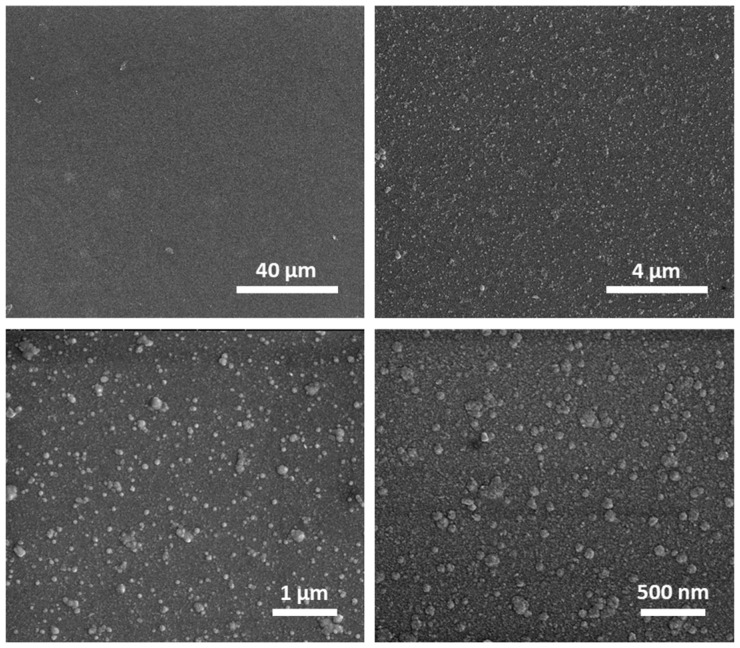
SEM images of the initial titania surface (sample R0) shown at different magnification.

**Figure 2:**
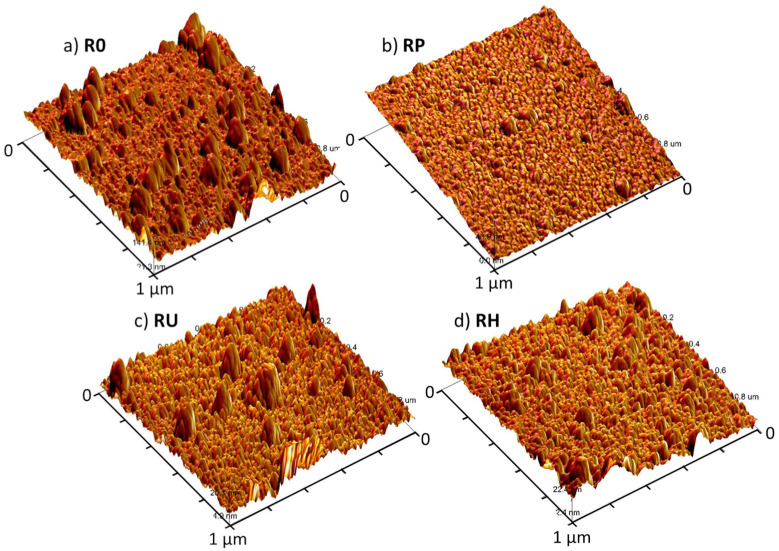
AFM images of the studied titania surfaces: intact R0 titania surface (**a**), RP oxygen plasma activated sample (**b**), RU sample activated with UV (**c**), and RH sample surface activated with H_2_O_2_ (**d**).

The SEM analysis of the bare titania surface (sample R0) under various magnifications was performed to examine the properties of the surface. As shown in Figure 1, the glass slide is covered with a continuous and tight titania film; however, at higher magnification, random particles or agglomerates of these particles with sizes up to 100 nm are visible. The analysis of the AFM images for the R0 sample also confirms the presence of particles/agglomerates present on the bare TiO_2_ surface—an exemplary photo of this sample is shown in Figure 2a.

In order to monitor the morphological changes resulting from the applied activation protocols, the AFM analysis was performed on the slides directly after activation process, i.e., for the oxygen plasma (RP), UV (RU), and H_2_O_2_ (RH) activated sample. Selected 3D-projected AFM photos are shown in Figure 2. The surface topography of the bare untreated slide is similar to projection captured on the SEM image. However, the surface topography changes significantly after plasma activation (Figure 2b), i.e., the surface is smoother, and only a small number of agglomerates/adherent particles can be observed, suggesting removal of the residual particles upon the surface treatment with oxygen plasma. In turn, the surface topography of the UV-activated sample RU (Figure 2c) looks quite like that of the initial non-activated sample R0 (Figure 2a). Interestingly, the RH surface activated with hydrogen peroxide (Figure 2d) shows topography with the intermediate pattern between the RP plasma-activated surface (Figure 2b) and the UV-activated surface (Figure 2c).

The discussed visual observations of morphological changes are confirmed by statistical analysis of the AFM roughness data (Table 1), which also reveals important differences. An arithmetical mean height (S_a_) and root mean square height (S_q_) are commonly accepted measures used to evaluate the surface roughness [34]. S_a_ expresses the averaged difference in the height of each point compared to the arithmetical mean of the surface. S_q_ represents the root mean square value of ordinate values and is equivalent to the standard deviation of the heights. The third parameter, S_diff_, is the percentage difference between the observed three-dimensional surface area and projected two-dimensional image area (in this case, 1 μm^2^). All three parameters have the highest mean values for the non-activated R0 surface (4.32 nm, 2.97 nm and 5.95%, respectively). Each treatment of the activation process results in a decrease in the mean value of all three parameters, and the level of the changes depends on the type of activation. For the surfaces RH and RP, the change of the mean values of the parameters is more pronounced when compared to the sample RU. The reason for this can be the fact that plasma and H_2_O_2_ activation are quite aggressive types of treatments, involving free radicals or active ions directly approaching the surface and provoking its modification. In contrast, during UV activation, no active chemical moieties capable of chemical reactivity with the surface are present directly. The active chemical forms occur in situ during the reaction of UV light with titania. This explains an effect of UV activation of the topmost layer available to UV light. The mechanism of these UV-initiated chemical changes was described elsewhere [35].

On a macroscopic scale, the changes resulting from the conducted activation processes were monitored by means of contact angle measurements. These measurements taken immediately after the activation were performed just after the processing (0 h) and after 24 h post-activation (24 h). The reason was to determine the wettability change after the activation process as well as the persistence of the increased hydrophilicity of the surface due to activation. The obtained results are summarized in Table 2.

**Figure 3:**
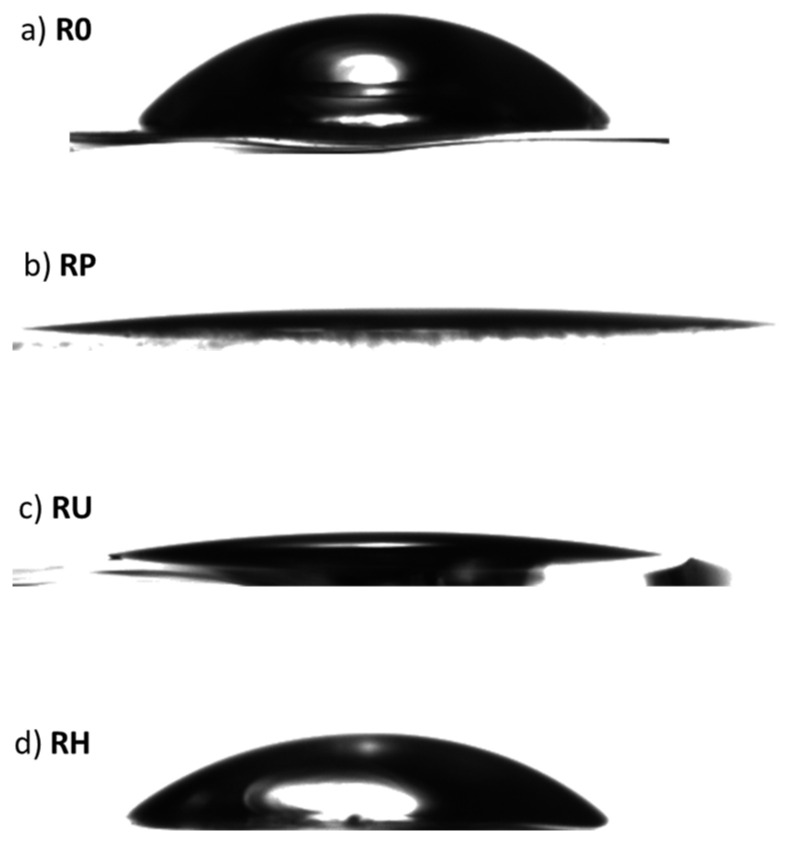
Wettability of the initial, untreated R0 titania surface (**a**) and the resulting RP titania surfaces directly after activation with oxygen plasma (**b**), RU samples activated with UV (**c**), and RH surface activated with H_2_O_2_ (**d**) as presented by the shape of the photographed water drop.

Looking at the mean values of the contact angle measured immediately after activation (0 h), it is clear that for each of the activation methods used, the contact angle decreased, although to a different extent (Figure 3). The least pronounced change was observed for the surface activated with H_2_O_2_, for which the contact angle decreased from 64.1° to 58.3°. However, for the other two activation methods, the change in the contact angle is more pronounced: for the RP surface, the observed contact angle is 10.1°, and for the RU surface, it reaches 9.4°. This means that the obtained RU and RP surfaces are completely wettable [36,37] (Figure 3b,c). Interestingly, the origin of superhydrophilicity of the surfaces RP and RU can be slightly different, which is indicated later in the manuscript when discussing XPS data.

It was noticed that after 24 h, the surface wettability decreased as manifested by increasing contact angles. Such behavior is expected, as an activated highly energetic titania surface is very sensitive to ubiquitous volatile organic compounds, and even in a few hours, the wettability of the surface might drastically change [38]. Then, adsorption-induced re-relaxation of the titania substrate can also take place [39]. Therefore, even small variations in the activation protocol, handling, and storage procedures of such activated surfaces might be responsible for the large deviations and final properties of the activated TiO_2_ surface.

Interestingly, after 24 h, the wettability of the RH surface (67.6°) is close to the wettability of the non-activated surface (65.7°), which means that H_2_O_2_ treatment provides the least stable surface activation. In the case of the other two treatments, the wettability after 24 h is much higher (39.9° for RP and 37.9° for RU) than in the case of the untreated surface (65.7°). When confronting wettability data with the AFM imaging data (with the greatest morphological changes on the RH surface), it is evident that the surface topography could not be alone responsible for the increase in surface wettability (hydrophilicity). Obviously, the chemical composition of the surface may also play a role, particularly the ability of each activation method to hydroxylate the surface.

Therefore, chemical compositions on the untreated and activated surfaces were examined using the XPS approach. The activation protocols were synchronized such that they were completed exactly at the same time and the freshly activated samples were immediately transferred to the XPS vacuum chamber for analysis upon reaching the appropriate low vacuum. The results of the XPS elemental analysis are presented in Table 3. Importantly, the studied samples coated with TiO_2_ film contained a significant amount of carbon that seems an integral component of the studied samples. The deconvolution pattern of the C1s signal is somehow typical for organic species.

The carbon content turned out to be a very convenient way of monitoring the effectiveness of activation. Its initial amount (33.3 at %) significantly decreased after the plasma activation (26.8 at %) and the process of UV irradiation (24.4 at %). On the other hand, the treatment with H_2_O_2_ had no effect on the carbon amount (34.7 at %). The reason for the most effective carbon elimination by UV radiation lies in a well-known photoinduced self-cleaning capability of titania due to the surface reorganization mechanism as follows [24]:

≡O–Ti–O≡ + H_2_O ↔ ≡Ti–OH + HO–Ti≡ (1)

This mechanism is confirmed by changes in the content of other elements, i.e., oxygen and titanium. There was a negative correlation between both elements and the surface carbon amount—the greater the decrease in the amount of carbon, the greater the increase in the amount of oxygen and titanium. For example, after plasma treatment, the carbon content is reduced from 33.3 at % to 26.8 at % and can be considered as an indicator of the efficiency of the cleaning process. At the same time, the oxygen content increased from 44.3 at % to 48.1 at %, and the titanium content increased from 18.0 at % to 20.3 at %. It turns out that H_2_O_2_ activation caused no loss of carbon from the surface as well as no significant increase in the amount of oxygen, unlike the other two activation methods (plasma and UV activation). These data are fully consistent with the surface wettability data obtained from the contact angle measurements and confirm the fact of the chemical changes in the surface that are mainly responsible for the increased hydrophilicity.

**Figure 4:**
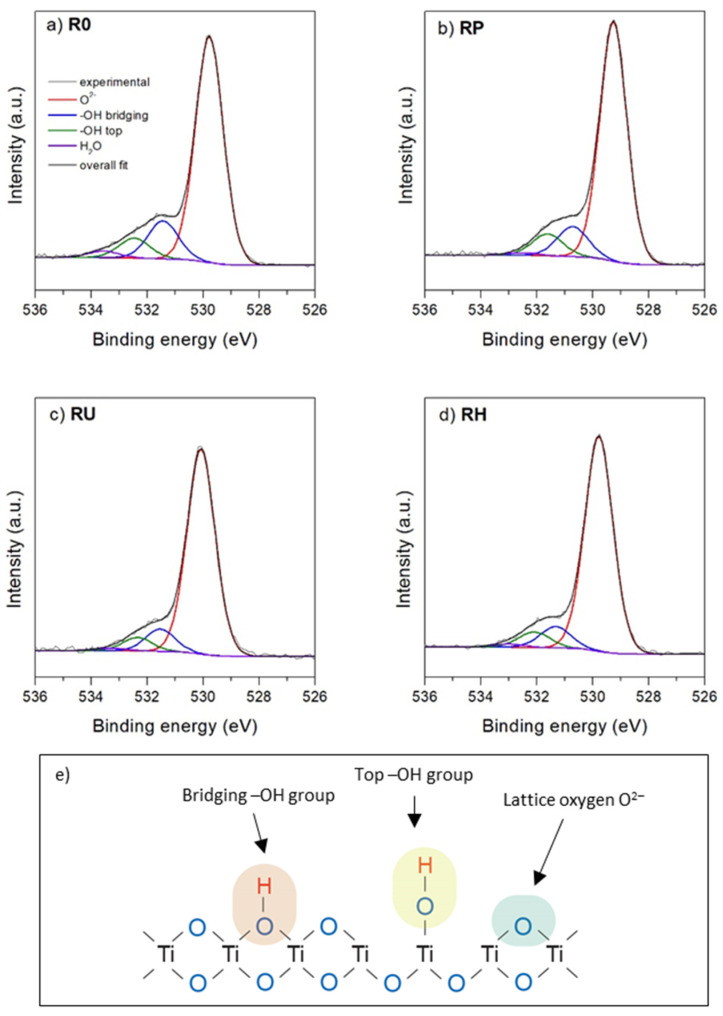
Deconvolution of O 1s core energy level for the studied titania surfaces: R0 unmodified titania surface (**a**), RP oxygen plasma treated (**b**), UV-activated RU sample (**c**), and RH sample H_2_O_2_-activated (**d**). The figure also shows various oxygen groups [43] for better interpretation of the XPS results (**e**).

Very interesting conclusions can be drawn from thorough analysis of the deconvoluted O 1s signal (the most indicative and most frequently discussed signal in the literature in relation to titania materials [33,40,41,42]). After Shirley background subtraction and deconvolution, the O 1s core energy level of the untreated R0 titania surface gives rise to four components, as shown in Figure 4a. The main component at 530.0 eV BE can be undoubtedly interpreted as originating from bulk O^2−^ oxygen atoms in titania lattice, which was always reported for TiO_2_ samples. The two other components should correspond to the surface groups. Two different OH groups have been reported in the literature for hydrated/activated titania surfaces: acidic hydroxyl groups linked to the bridging oxygens and basic hydroxyl groups bonded to five-coordinated Ti^4+^ cations. Indeed, the existence of the two next components shifted ≈1.6 eV and ≈2.9 eV compared to the main bulk peak is observed. These shifted components can be attributed to the two types of hydroxyl groups on the titania surface: (i) a two-fold bridging −OH group with acidic character (shifted 1.6 eV) and (ii) a single-fold ones “top” −OH group with basic character (shifted ≈2.9 eV) [43,44]. The weakest contribution at ≈533.6 eV can be tentatively attributed to the oxygen of molecularly physisorbed water in the upper hydration layer [33].

The activation protocols employed in this paper lead to significant changes in the intensity of the individual components of the O 1s core energy level. As discussed earlier, protocols for plasma and UV activation led to a significant increase in the oxygen content, but importantly, the relative amounts of individual surface oxygen groups also changed. For a plasma-activated sample, the relative shares of bulk O^2−^ oxygen atoms increased (from 33.8 at % to 38.2 at %), and the number of bridging hydroxyl groups and top hydroxyl groups are also changed (from 6.1 at.% to 5.5 at.% and from 3.3 at % to 4.0 at %, respectively).

Activation by UV irradiation leads to a slight reduction in the number of hydroxyl groups while significantly increasing the amount of bulk O^2−^ oxygen atoms (from 33.8 at % to 41.6 at %) due to the most effective surface cleaning (which is also confirmed by the most pronounced reduction in the amount of carbon on the surface). In the case of the H_2_O_2_-activated sample, the relative share of bulk O^2−^ oxygen atoms increased slightly (from 33.8 at % to 35.6 at %), while the content of both types of hydroxyl groups decreased significantly (from 6.1 at % to 3.8 at % and from 3.3 at % to 2.8 at %, for bridging and top hydroxyl groups, respectively). Comparing the above results, it is evident that plasma is most effective in generating stable surface hydroxyl groups of acidic (bridging −OH) and basic nature (top −OH). Moreover, it is worth noting the difference between plasma and UV radiation with regard to the hydrophilicity of the surface. In the case of plasma, the hydrophilicity increases rather due to the large number of hydroxyl groups (especially basic top −OH groups), while in the case of UV irradiation, the main reason is the surface cleaning effect. This effect is clearly confirmed by the greatest loss of carbon and the increase in oxygen and titanium on the surface under the influence of UV radiation. Finally, it should be noted that due to the fact that for some applications, the presence of either acidic or basic carboxyl groups may be required, the selection of the appropriate activation protocol can be of a huge importance. It was reported that the type of surface hydroxyls could significantly modulate the surface property affecting the adsorption mode of studied adsorbents and promote the photocatalytic efficiency [13,45,46]. Therefore, the possibility of influencing amounts of acid and basic hydroxyl groups on the TiO_2_ surface makes the appropriate activation method an important tool for controlling the final properties of this material. For example, plasma activation can decrease the surface acidity by increasing the relative share of basic hydroxyl groups on the surface.

## 4. Conclusions

Three popular methods of surface activation of the titania surface were compared in a comprehensive and detailed manner for the first time. Each of them resulted in a titania surface with different properties, both in terms of surface morphology and, above all, hydrophilicity and surface chemistry. Oxygen plasma and UV activation were the most effective, which is clearly indicated by the complete wetting of the resulting surfaces (contact angles: 10.1° and 9.4°, respectively) and significant changes in the surface chemistry (much lower carbon amount, significantly higher oxygen and noticeably higher titanium content). On the other hand, H_2_O_2_ activation was shown to be the least effective procedure—it led to only a slight reduction of wettability (from 64.1° to 41.3°) and minor changes in the surface chemistry. As shown in this work, hydrophilicity appears to depend mainly on the resulting surface chemistry, but the effect of surface morphology must also be considered. Consequently, our results show that the choice of the activation method of the titania coated surface might play a key role in the subsequent applications of the activated surfaces/materials.

## Figures and Tables

**Table 1:** Analysis of surface roughness data derived from statistical processing of AFM images of the samples subjected to different activation protocols.

Sample	Method of Activation	AFM Roughness Data		
		S_q_ (nm)	S_a_ (nm)	S_diff_ (%)
R0	untreated	4.32 ± 0.3	2.97 ± 0.23	5.95 ± 0.44
RP	plasma	3.97 ± 1.40	2.57 ± 0.55	5.24 ± 0.18
RU	UV	4.11 ± 0.84	2.73 ± 0.57	5.56 ± 0.16
RH	H_2_O_2_	3.15 ± 0.36	2.27 ± 0.25	5.34 ± 0.19

**Table 2:** Values of contact angle of the untreated and activated titania surface.

Sample	Method of Activation	Contact Angle (Degrees)	
		0 h	After 24 h
R0	untreated	65.7 ± 2.8	65.7 ± 2.8
RP	plasma	10.1 ± 1.5	39.9 ± 3.1
RU	UV	9.4 ± 2.1	37.9 ± 1.9
RH	H_2_O_2_	58.3 ± 1.2	67.6 ± 2.1

**Table 3:** XPS elemental composition and contact angle measurements of the untreated and activated titania surface.

Sample	Method of Activation	XPS Elemental Composition (at %) *				Oxygen Surface Species (at %)			
		%C	%O	%Ti	Ti:O Ratio	O^2−^	−OH (Bridging)	−OH (top)	Water
R0	untreated	33.3 ± 3.0	44.3 ± 2.2	18.0 ± 0.9	0.41	33.8	6.1	3.3	1.1
RP	plasma	26.8 ± 2.4	48.1 ± 1.8	20.3 ± 0.8	0.42	38.2	5.5	4.0	0.5
RU	UV	24.4 ± 3.1	49.8 ± 2.2	21.3 ± 0.9	0.43	41.6	4.7	2.9	0.5
RH	H_2_O_2_	34.7 ± 2.8	42.9 ± 2.1	19.2 ± 0.9	0.45	35.6	3.8	2.8	0.6

* minor amount of other elements were also detected, i.e., N, F, Na, Ca.

## Data Availability

The data presented in this study are available on request from the corresponding author.

## References

[1] Blanchart P. (2018). Extraction, Properties and Applications of Titania. Industrial Chemistry of Oxides for Emerging Applications.

[2] Haider A.J., Jameel Z.N., Al-Hussaini I.H.M. (2019). Review on: Titanium Dioxide Applications. Energy Procedia.

[3] Chen X., Mao S.S. (2007). Titanium dioxide nanomaterials: Synthesis, properties, modifications and applications. Chem. Rev..

[4] Carp O., Huisman C.L., Reller A. (2004). Photoinduced reactivity of titanium dioxide. Prog. Solid State Chem..

[5] Fujishima A., Rao T.N., Tryk D.A. (2000). Titanium dioxide photocatalysis. J. Photochem. Photobiol. C Photochem. Rev..

[6] Adachi T., Latthe S.S., Gosavi S.W., Roy N., Suzuki N., Ikari H., Kato K., Katsumata K.I., Nakata K., Furudate M. (2018). Photocatalytic, superhydrophilic, self-cleaning TiO_2_ coating on cheap, light-weight, flexible polycarbonate substrates. Appl. Surf. Sci..

[7] Pantaroto H.N., Cordeiro J.M., Pereira L.T., de Almeida A.B., Nociti Junior F.H., Rangel E.C., Azevedo Neto N.F., da Silva J.H.D., Barão V.A.R. (2021). Sputtered crystalline TiO_2_ film drives improved surface properties of titanium-based biomedical implants. Mater. Sci. Eng. C.

[8] Jafari S., Mahyad B., Hashemzadeh H., Janfaza S., Gholikhani T., Tayebi L. (2020). Biomedical Applications of TiO_2_ Nanostructures: Recent Advances. Int. J. Nanomedicine.

[9] Bronze-Uhle E.S., Dias L.F.G., Trino L.D., Matos A.A., de Oliveira R.C., Lisboa-Filho P.N. (2019). Physicochemical characterization of albumin immobilized on different TiO_2_ surfaces for use in implant materials. Colloids Surf. A Physicochem. Eng. Asp..

[10] Diebold U. (2003). The Surface Science of Titanium Dioxide. Surf. Sci. Rep..

[11] Mino L., Morales-García Á., Bromley S.T., Illas F. (2021). Understanding the nature and location of hydroxyl groups on hydrated titania nanoparticles. Nanoscale.

[12] Nanayakkara C.E., Larish W.A., Grassian V.H. (2014). Titanium dioxide nanoparticle surface reactivity with atmospheric gases, CO_2_, SO_2_, and NO_2_: Roles of surface hydroxyl groups and adsorbed water in the formation and stability of adsorbed products. J. Phys. Chem. C.

[13] Wu C.Y.C.H., Tu K.J., Deng J.P., Lo Y.S., Wu C.Y.C.H. (2017). Markedly Enhanced Surface Hydroxyl Groups of TiO_2_ Nanoparticles with Superior Water-Dispersibility for Photocatalysis. Materials.

[14] Pan L., Zou J.J., Zhang X., Wang L. (2011). Water-mediated promotion of dye sensitization of TiO_2_ under visible light. J. Am. Chem. Soc..

[15] Jo M.R., Yu J., Kim H.J., Song J.H., Kim K.M., Oh J.M., Choi S.J. (2016). Titanium Dioxide Nanoparticle-Biomolecule Interactions Influence Oral Absorption. Nanomaterials.

[16] Sampath J., Kullman A., Gebhart R., Drobny G., Pfaendtner J. (2020). Molecular recognition and specificity of biomolecules to titanium dioxide from molecular dynamics simulations. Npj Comput. Mater..

[17] Sano K.I., Shiba K. (2003). A Hexapeptide Motif that Electrostatically Binds to the Surface of Titanium. J. Am. Chem. Soc..

[18] Li Y., Song Y., Ma A., Li C. (2019). Surface immobilization of TiO_2_ nanotubes with bone morphogenetic protein-2 synergistically enhances initial preosteoblast adhesion and osseointegration. Biomed Res. Int..

[19] Nucia A., Tomczyńska-Mleko M., Okoń S., Kowalczyk K., Terpiłowski K., Pérez-Huertas S., Nishinari K., Nastaj M., Mleko S. (2021). Surface properties of gluten deposited on cold plasma-activated glass. Food Hydrocoll..

[20] Wiącek A.E., Gozdecka A., Jurak M., Przykaza K., Terpiłowski K. (2018). Wettability of plasma modified glass surface with bioglass layer in polysaccharide solution. Colloids Surf. A Physicochem. Eng. Asp..

[21] Otitoju T.A., Ahmad A.L., Ooi B.S. (2017). Superhydrophilic (superwetting) surfaces: A review on fabrication and application. J. Ind. Eng. Chem..

[22] Bharti B., Kumar S., Lee H.N., Kumar R. (2016). Formation of oxygen vacancies and Ti^3+^ state in TiO_2_ thin film and enhanced optical properties by air plasma treatment. Sci. Rep..

[23] Wang J., Lin Z. (2010). Dye-sensitized TiO^2^ nanotube solar cells with markedly enhanced performance via rational surface engineering. Chem. Mater..

[24] Mills A., Crow M. (2008). A study of factors that change the wettability of titania films. Int. J. Photoenergy.

[25] Stevens N., Priest C.I., Sedev R., Ralston J. (2003). Wettability of Photoresponsive Titanium Dioxide Surfaces. Langmuir.

[26] Carretero-Genevrier A., Boissiere C., Nicole L., Grosso D. (2012). Distance dependence of the photocatalytic efficiency of TiO^2^ revealed by in situ ellipsometry. J. Am. Chem. Soc..

[27] Masood M.T., Weinberger C., Qudsia S., Rosqvist E., Sandberg O.J., Nyman M., Sandén S., Vivo P., Aitola K., Lund P.D. (2019). Influence of titanium dioxide surface activation on the performance of mesoscopic perovskite solar cells. Thin Solid Films.

[28] Giammaria G., Van Rooij G., Lefferts L. (2019). Plasma Catalysis: Distinguishing between Thermal and Chemical Effects. Catalysts.

[29] Jackson M.J., Waqar A. (2007). Surface Engineered Surgical Tools and Medical Devices.

[30] Curci R., Edwards J.O. Activation of Hydrogen Peroxide by Organic Compounds. https://link.springer.com/chapter/10.1007/978-94-017-0984-2_3.

[31] Boonstra A.H., Mutsaers C.A.H.A. (2002). Adsorption of hydrogen peroxide on the surface of titanium dioxide. J. Phys. Chem..

[32] Chouirfa H., Bouloussa H., Migonney V., Falentin-Daudré C. (2019). Review of titanium surface modification techniques and coatings for antibacterial applications. Acta Biomater..

[33] Perron H., Vandenborre J., Domain C., Drot R., Roques J., Simoni E., Ehrhardt J.J., Catalette H. (2007). Combined investigation of water sorption on TiO_2_ rutile (1 1 0) single crystal face: XPS vs. periodic DFT. Surf. Sci..

[34] Barczak M., Bandosz T.J. (2019). Evaluation of nitrogen- and sulfur-doped porous carbon textiles as electrode materials for flexible supercapacitors. Electrochim. Acta.

[35] Unosson E., Welch K., Persson C., Engqvist H. (2013). Stability and prospect of UV/H_2_O_2_ activated titania films for biomedical use. Appl. Surf. Sci..

[36] Drelich J., Chibowski E. (2010). Superhydrophilic and Superwetting Surfaces: Definition and Mechanisms of Control. Langmuir.

[37] Drelich J., Chibowski E., Meng D.D., Terpilowski K. (2011). Hydrophilic and superhydrophilic surfaces and materials. Soft Matter.

[38] Kanta A., Sedev R., Ralston J. (2005). Thermally- and Photoinduced Changes in the Water Wettability of Low-Surface-Area Silica and Titania. Langmuir.

[39] Silber D., Kowalski P.M., Traeger F., Buchholz M., Bebensee F., Meyer B., Wöll C. (2016). Adsorbate-induced lifting of substrate relaxation is a general mechanism governing titania surface chemistry. Nat. Commun..

[40] Benkoula S., Sublemontier O., Patanen M., Nicolas C., Sirotti F., Naitabdi A., Gaie-Levrel F., Antonsson E., Aureau D., Ouf F.X. (2015). Water adsorption on TiO_2_ surfaces probed by soft X-ray spectroscopies: Bulk materials vs. isolated nanoparticles. Sci. Rep..

[41] Bullock E.L., Patthey L., Steinemann S.G. (1996). Clean and hydroxylated rutile TiO_2_(110) surfaces studied by X-ray photoelectron spectroscopy. Surf. Sci..

[42] Sham T.K., Lazarus M.S. (1979). X-ray photoelectron spectroscopy (XPS) studies of clean and hydrated TiO_2_ (rutile) surfaces. Chem. Phys. Lett..

[43] Giannakoudakis D.A., Farahmand N., Łomot D., Sobczak K., Bandosz T.J., Colmenares J.C. (2020). Ultrasound-activated TiO_2_/GO-based bifunctional photoreactive adsorbents for detoxification of chemical warfare agent surrogate vapors. Chem. Eng. J..

[44] Giannakoudakis D.A., Qayyum A., Łomot D., Besenhard M.O., Lisovytskiy D., Bandosz T.J., Carlos Colmenares J. (2021). Boosting the Photoactivity of Grafted Titania: Ultrasound-Driven Synthesis of a Multi-Phase Heterogeneous Nano-Architected Photocatalyst. Adv. Funct. Mater..

[45] Luisa M., Gioia D., Martins L.M., Pastor I.M., Rychtowski P., Tryba B., Skrzypska A., Felczak P., Sré Nscek-Nazzal J., Wróbel R.J. (2022). Role of the Hydroxyl Groups Coordinated toTiO_2_ Surface on the Photocatalytic Decomposition of Ethylene at Different Ambient Conditions. Catalysts.

[46] Li W., Du D., Yan T., Kong D., You J., Li D. (2015). Relationship between surface hydroxyl groups and liquid-phase photocatalytic activity of titanium dioxide. J. Colloid Interface Sci..

